# Recurrent tick bites induce high IgG1 antibody responses to α‐Gal in sensitized and non‐sensitized forestry employees in Luxembourg

**DOI:** 10.1002/clt2.12396

**Published:** 2024-10-13

**Authors:** Neera Chakrapani, Kyra Swiontek, Judith M. Hübschen, Jörg Fischer, Maria Ruiz‐Castell, Francoise Codreanu‐Morel, Farah Hannachi, Martine Morisset, Markus Ollert, Annette Kuehn, Claude P. Muller, Christiane Hilger

**Affiliations:** ^1^ Department of Infection and Immunity Luxembourg Institute of Health Esch‐sur‐Alzette Luxembourg; ^2^ Faculty of Science, Technology and Medicine University of Luxembourg Esch‐sur‐Alzette Luxembourg; ^3^ Faculty of Medicine Department of Dermatology Eberhard Karls University of Tübingen Tübingen Germany; ^4^ Department of Precision Health Luxembourg Institute of Health Strassen Luxembourg; ^5^ The Immunology–Allergology Unit Center Hospitalier Luxembourg Luxembourg Luxembourg; ^6^ Department of Dermatology and Allergy Center Odense Research Center for Anaphylaxis University of Southern Denmark Odense Denmark; ^7^ Present address: Dermatology & Allergology University Hospital Augsburg Germany.; ^8^ Present address: Allergy Unit Angers University Hospital Angers France.; ^9^ Present address: ALK‐Global research Hoersholm Denmark.

**Keywords:** α‐Gal IgG, alpha‐gal syndrome, high‐risk population, sensitization, specific IgE

## Abstract

**Background:**

The α‐Gal syndrome (AGS) is characterized by the presence of specific IgE‐antibodies to the carbohydrate galactose‐α‐1,3‐galactose (α‐Gal). Sensitization to α‐Gal has been associated with tick bites and individuals exposed to ticks have an elevated risk of sensitization. The aim of this study was to analyze IgG and IgE antibody responses to α‐Gal in a high‐risk cohort of forestry employees (FE) in Luxembourg.

**Methods:**

Questionnaires and serum samples of FE from Luxembourg (*n* = 219) were retrospectively analyzed. α‐Gal specific IgE was quantified by ImmunoCAP, α‐Gal specific IgG and subclasses IgG_1–4_ were determined by ELISA. Additionally, sera from population‐based controls (*n* = 150) and two groups of food‐allergic patients, patients with AGS (*n* = 45) and fish‐allergic patients (*n* = 22) were assessed for IgG antibody responses to α‐Gal and cod extract.

**Results:**

Twenty‐one percent of FE was sensitized to α‐Gal (sIgE ≥ 0.1 kU_A_/L). Both sensitized and non‐sensitized FE exhibited high levels of α‐Gal specific IgG, IgG1 and IgG3 compared with controls, indicating a stimulation of IgG responses by recurrent tick bites, independent of the sensitization status. AGS patients had the highest levels of IgG1 and IgG2 antibodies, whereas the profile of fish‐allergic patients was similar to the profile of the controls for which anti‐α‐Gal responses were dominated by IgG2 antibodies. α‐Gal sIgG4 levels were either very low or undetectable in all groups.

**Conclusion:**

Our study provides evidence for a continuous stimulation of α‐Gal related immune responses by repeated tick bites, translating into highly elevated levels of IgG1 antibodies directed against α‐Gal.

## 1 INTRODUCTION

The α‐Gal syndrome (AGS) is a novel form of a tick‐borne disease characterized by a delayed onset of allergic reaction usually within 2–6 h after consumption of mammalian meat and by‐products.^1^ Numerous cases of this form of red meat allergy aka α‐Gal allergy have been reported in Europe and the US, and cases are emerging in Africa, South America, and Asia as well.^2^, ^3^, ^4^ All mammals naturally express the carbohydrate galactose‐α‐1,3‐galactose (α‐Gal) on glycoprotein and glycolipid structures, except humans, apes, and old‐world monkeys, due to a mutation which resulted in inactivation of the α‐1,3‐galactosyltransferase (α‐1,3‐GT) gene thereby abolishing α‐Gal expression.^1^ Owing to this, humans produce anti‐α‐Gal antibodies upon antigenic stimulation, which presumably provide an evolutionary advantage by playing a protective role against pathogens.^5^ Consequently, anti‐α‐Gal IgG, IgM, and IgA antibodies have been identified in human serum^6^ and IgA in secretions such as saliva, milk, vaginal secretion etc..^7^ These antibodies are present in abundant quantities; in addition to their protective role they can also be detrimental in certain cases such as rejection of mammalian grafts in humans.^8^

Clinical observation in humans as well as tick feeding on α‐GALT‐KO mice has clearly shown induction of α‐Gal‐specific IgE by tick bites.^9^, ^10^, ^11^, ^12^ Therefore, frequent exposure to tick bites can possibly increase the chances of sensitization. In this context, various factors contributing to increased exposure such as occupation, geographical location, and recreational activities were found to correlate with increased frequency of tick bites and sensitization to α‐Gal.^13^, ^14^, ^15^, ^16^, ^17^, ^18^ Since α‐Gal is immunogenic in humans,^19^ it is of interest to investigate the potential induction of specific IgG in response to tick bites. In previous studies, AGS patients were found to possess higher levels of α‐Gal specific IgG compared to healthy individuals or patients with a non‐meat related allergy,^20^, ^21^, ^22^ furthermore, they were found to produce higher levels of α‐Gal IgG1^20^, whereas in commercial pharmaceutical human immunoglobulin preparations α‐Gal IgG2 was the dominant subclass.^23^, ^24^ This finding was confirmed in healthy blood donors.^25^ However, there is a lack of data on α‐Gal IgG induction in tick exposed sensitized and non‐sensitized individuals, which can help shed more light on immune responses towards α‐Gal.

In the present study, we investigated α‐Gal specific IgG and IgE responses among forestry employees (FE) in Luxembourg, a group with high tick exposure, and determined various demographic factors and their impact on IgE sensitization. In addition, as previous studies already showed increased levels of α‐Gal IgG in AGS patients, we further analyzed levels of α‐Gal IgG and IgG subclasses in a group reporting frequent tick bites and in a general population group. Our study results confirm a direct role of tick bites in increased IgG levels to α‐Gal. Furthermore, we identified distinct (allergen‐specific) IgG subclass profiles when comparing anti‐α‐Gal IgG antibodies in AGS patients to anti‐cod antibodies in a group of patients with protein related food allergies.

## 2 METHODS

### 2.1 Study population

#### 2.1.1 Forestry employees

FE were recruited in 2012 and 2013 in collaboration with the Nature and Forest Agency in Luxembourg in the frame of a study on Lyme disease (MarLyBor, Ethical approval CNER N°201111/07). All participants gave their informed consent for participation in the study. Data on hours spent outside, years of occupation as forestry workers, number of tick bites the year before recruitment, date of last tick bite, sex and age were recorded from participants during an interview conducted by a member of the study team. Only participants for whom serum samples were available were included in the present study (*n* = 219). Serum samples were collected in May 2012 and April 2013. Clinical data were not recorded during initial recruitment and such data could not be obtained retrospectively from participants as they did not give their consent to be recontacted.

#### 2.1.2 Patients

Sera from symptomatic AGS patients previously recruited at the outpatient clinic of the National Immunology‐Allergology Unit at the Centre Hospitalier de Luxembourg (*n* = 21) and the Allergology Unit of the Department of Dermatology of Eberhard Karls University in Tubingen (*n* = 24) were included in the study. The majority of those patients were subject of a previous publication,^26^ 12 patients were newly recruited. All patients had a positive clinical history of α‐Gal allergy and were previously tested for total IgE and specific IgE (sIgE) levels to α‐Gal (sIgE ≥0.35 kU_A_/L) (ImmunoCAP; Thermo Fisher Scientific, Uppsala, Sweden). In addition, sera from fish‐allergic patients with a clinical history of allergy to fish (*n* = 22; sIgE to cod ≥0.35 kU_A_/L, positive prick‐to‐prick test >3 mm) were included as a food‐allergic control population. The study was approved by the national committee for medical research ethics in Luxembourg (201,307/04, 201,605/03 and 201,910/04) and by the ethics commission of the University Medical Faculty in Tubingen (158/2016BO1). Written informed consent was obtained from all study participants.

#### 2.1.3 Control population

The control subjects were part of the European Health Examination Survey, Luxembourg (EHES‐LUX) (Ethical approval 201,205/07‐SU1).^27^ A sample of 150 subjects was drawn from this cohort to select a control cohort for this study that was age‐matched with the FE cohort, gender balanced and with an allergic sensitization prevalence of 44%, which reflects the prevalence determined by Czolk et al^28^ for the representative population sample (*n* = 1462).

### 2.2 Specific and total IgE quantification

sIgE to α‐Gal were measured by ImmunoCAP (Thermo Fisher Scientific, Uppsala, Sweden) as per the manufacturer's standard procedure. Total IgE values were measured using a human‐IgE ELISA kit (Invitrogen, Thermo Fisher Scientific, Austria) following the manufacturer's instructions. Total IgE values were calculated by extrapolating OD values from the standard curve.

sIgE to cod allergens was measured using an in‐house ELISA^29^ with coated codfish flesh extract. Briefly, 0.5 gm per mL of codfish flesh was mixed with single detergent lysis buffer (50 mM Tris‐HCl, 150 mM NaCl, pH 8) with 1% Triton‐X100, homogenized for 10 min at 30 Hz with 5 mm steel bead per 2 mL tube in Retsch Mill and centrifuged for 30 min at 17.500 × g. The supernatant was heated for 10 min at 95°C, centrifuged for 15 min at 17.500 × g, and the protein concentration was measured with Bradford's assay. Next, ELISA plates (Maxisorp, Nunc, Thermo Fisher Scientific, Denmark) were coated overnight with the heated codfish extract at 5 μg/mL in phosphate buffered saline (PBS), followed by the in‐house ELISA procedure for sIgE detection.^29^

### 2.3 IgG subclass phenotyping by enzyme‐linked immunosorbent assay

For α‐Gal IgG subtyping, 384‐well ELISA plates (high‐binding, Greiner Bio‐one, Germany) were coated with α‐Gal coupled to human serum albumin (α‐Gal HSA, Dextra Labs, Reading, UK) at 2 μg/mL in PBS overnight at 4°C. After blocking the wells with 1% HSA/TBST (Tris buffered saline with 0.05% Tween‐20), sera were diluted in 0.5% HSA/TBST (1/50 for IgG determination and 1/20 for subclasses IgG1, IgG2, IgG3, and IgG4) and added to the wells for 2 h (300 rpm/room temperature). As IgG1 levels were very high in patients with AGS and FE, a serum dilution of 1/50 was preferred for the generation of the data used for the correlation analysis between IgG1 and sIgE levels. For detection, alkaline phosphatase (AP) labeled anti‐IgG or anti‐IgG1 were diluted 1/2000, or anti‐IgG2, anti‐IgG3 and anti‐IgG4 were diluted 1/500 in 0.5% HSA/TBST and incubated for 2 h (300 rpm/room temperature). Signal was developed with para‐nitrophenyl phosphate solution (Sigmafast, Sigma Aldrich) and optical density (OD) read at 405 nm on Spectramax 384Plus ELISA reader (Molecular Devices, UK). Serum with high IgG levels to α‐Gal was used as a reference across plates to minimize inter‐plate variability. All antibodies were purchased from Southern‐Biotech, UK (9040‐04, 9054‐04, 9070‐04, 9210‐04, 9200‐04). Antibodies were tested for a potential cross‐reactivity among IgG subclasses and found to be highly specific for the respective IgG1, IgG2, IgG3 or IgG4 subclasses (data not shown).

For a more sensitive detection of IgG4, coated and blocked ELISA plates as described above were incubated overnight with sera diluted 1/10. Detection was performed the following day with biotin‐labeled anti‐IgG4 (Southern Biotech) by incubating for 90 min, followed by streptavidin‐AP (BD Biosciences) for 30 min. The signal was measured as mentioned above.

### 2.4 Statistical analysis

IgE and IgG data were analyzed in GraphPad Prism v9 (GraphPad Software, La Jolla, California). Significance of demographic factors in sensitized versus non‐sensitized groups was compared with the Chi‐squared/Fischer's exact test. Differences in IgG subclass levels between the two groups were determined by multiple Mann–Whitney tests with the Holm‐Šídák method, setting the family wise alpha threshold to 0.05. The significance of variation in antibody levels across groups was compared using the Kruskal–Wallis with Dunn's multiple comparison test.

## 3 RESULTS

### 3.1 High α‐Gal sIgE prevalence among forest service employees in Luxembourg

**FIGURE 1:**
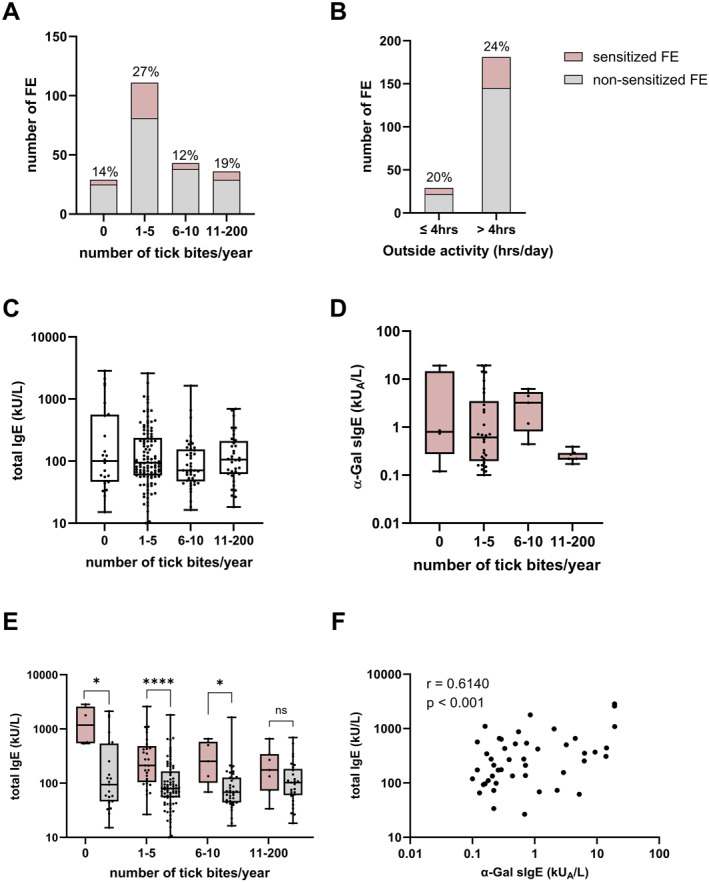
Sensitization prevalence to α‐Gal among FE. (A) Sensitization prevalence among FEs in percentage per category of annual tick bites (Fischer's exact test not significant). (B) Sensitization prevalence among FE in percentage per category of daily outdoor activity expressed in hours (chi square test not significant). (C) Total IgE levels in the FE cohort per number of annual tick bites (Kruskal‐Wallis test not significant). (D) Specific IgE levels to α‐Gal in sensitized FE per number of annual tick bites (Kruskal‐Wallis test not significant). (E) Total IgE levels in sensitized and non‐sensitized FE per number of annual tick bites. Multiple Mann–Whitney comparison of differences in antibody levels between groups (*p < 0.05, ****p < 0.0001, ns: not significant). (F) Correlation between total IgE and α‐Gal sIgE levels in sensitized FE (Spearman *r* = 0.6140, *p* < 0.001). Whiskers extend to minimum and maximum values, median displayed as a line within each box. FE, forestry employees.

FE from Luxembourg were recruited in late spring 2012 and 2013, and data on age, sex, years in occupation, type of work, activity outside, history and number of tick bites were obtained at the inclusion visit (see demographic data, Table 1). The vast majority of participants were male (96%) and the median age of the employees was 44.5 years at the time of sample collection. Forty‐eight percent of the employees were employed for more than 20 years. Overall 89% employees reported tick‐bites each year. In total, 175 employees could recall the time of the last bite, with 82% of them reporting bites during the year preceding sample collection. More than half (111/195) of the employees reporting bites experienced between 1–5 tick‐bites on average (Table 1, Figure 1A).

**TABLE 1:** Demographic data of forest employees in Luxembourg.

	Total cohort	Workers	Rangers	Occupation not defined	Sensitized (sIgE α‐gal ≥ 0.1 kUᴀ/L)	Non‐sensitized (sIgE α‐gal < 0.1 kUᴀ/L)	Chi square/ Fischer's exact test (sensitized vs. non‐sensitized)
Number (%)	219	167	42	10	46 (21%)	173 (79%)	–
Sex (male %)	211/219 (96%)	162/169 (96%)	39/42 (93%)	10/10	45/46 (98%)	166/173 (96%)	–
Age in yrs (median; range)	44.5 (19–63)	45 (19–63)	41 (23–60)	47 (24–51)	43 (19–63)	45 (19–63)	–
Activity outside (hrs/day)							
≤4	29/211 (14%)	4/161	25/42	na	7/43 (16%)	22/168 (13%)	ns
>4	182/211 (86%)	157/161	17/42	8/8	36/43 (84%)	146/168 (87%)	
Years in occupation							
<10	59/205 (29%)	45/160	14/40	na	13/41 (32%)	46/164 (28%)	ns
10–20	48/205 (23%)	38/160	9/40	1/5	11/41 (27%)	37/164 (23%)	
>20	98/205 (48%)	77/160	17/40	4/5	17/41 (41%)	81/164 (49%)	
No. of tick bites/year^a^							
0	24/219 (11%)	18/167	5/42	1/10	4/46 (9%)	20/173 (12%)	ns
1–5	111/219 (50%)	84/167	22/42	5/10	30/46 (65%)	81/173 (47%)	
6–10	43/219 (20%)	33/167	8/42	2/10	5/46 (11%)	38/173 (22%)	
>10	36/219 (16%)	27/167	7/42	2/10	7/46 (15%)	29/173 (17%)	
Last tick bite							
Within 1 year of collection	143/175 (82%)	119/139	31/33	3/3	33/36 (92%)	120/139 (86%)	ns
>1 year	22/175 (13%)	20/139	2/33	na	3/36 (8%)	19/139 (14%)	

*Note*: The data are shown as absolute numbers (n/N). Total count (N) shows number of forestry employees that answered the question.

Abbreviations: hrs/day, no. of hours per day; na, not applicable; ns, not significant; yrs, years.

^a^ Number of tick bites/year reports the number of bites experienced annually.

Twenty‐one percent (*n* = 46) of the employees were sensitized against α‐Gal (sIgE ≥0.1 kU_A_/L), of those 57% (*n* = 26) had sIgE levels of ≥0.35 kU_A_/L. Ninety‐two percent of the sensitized and 86% of the non‐sensitized employees reported tick bites within 1 year of sample collection (Table 1); 65% of the sensitized participants reported between 1–5 tick bites whereas 47% of non‐sensitized individuals reported similar number of tick bites (Table 1). Sensitization prevalence was higher in those reporting 1‐5 yearly tick bites, but the difference was not significant (Figure 1A). A majority of employees (86%) reported working more than 4 h/day outside, although the prevalence of sIgE against α‐Gal was not associated with outdoor activity of more than 4 h/day (Table 1, Figure 1B). Total IgE levels and α‐Gal sIgE levels did not significantly vary with the number of tick bites (Figure 1C,D). Sensitized FE had in general higher total IgE levels than non‐sensitized participants (Figure 1E) and levels of total IgE and α‐Gal sIgE were correlated in sensitized employees (Spearman *r* = 0.6140, *p*‐value <0.001) (Figure 1F). Lastly, 35/162 non‐sensitized FE reported a history of Lyme disease, whereas 6/41 sensitized FE reported Lyme disease. The difference between sensitized versus non‐sensitized FE was not significant, which is in line with a previous study where no correlation between a history of Lyme disease and sensitization to α‐Gal was observed.^30^ Due to the lack of clinical data associated with this retrospective study, the number of FEs with symptoms upon ingestion of red meat could not be assessed.

### 3.2 α‐Gal IgG and IgG_1‐3_ subclass levels are higher among sensitized FE

**FIGURE 2:**
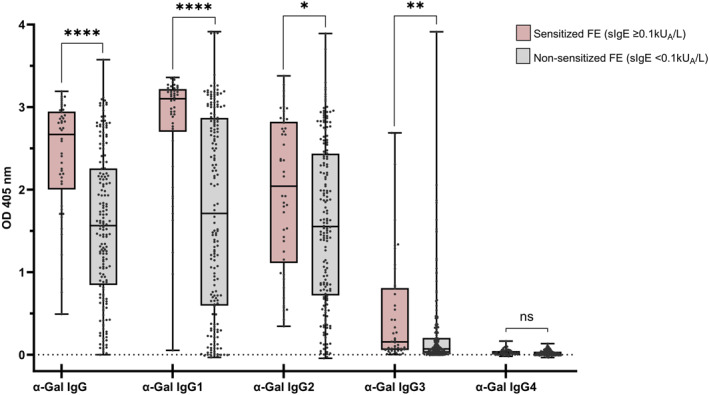
Sensitized FE produced significantly higher quantities of anti‐α‐Gal IgG. Box plot showing levels of anti‐α‐Gal IgG/subclasses in sensitized (α‐Gal‐specific IgE ≥ 0.1 kUᴀ/L) and non‐sensitized FE (*x*‐axis: IgG subclass, *y*‐axis: optical density (OD) at 405 nm). Multiple Mann–Whitney comparison of differences in antibody levels between groups (*p < 0.05, **p < 0.01, ****p < 0.0001, ns: not significant). Whiskers extend to minimum and maximum values, median displayed as a line within each box. FE, forestry employees.

Humans naturally produce anti‐α‐Gal IgG antibodies, but significantly higher IgG levels were described in AGS patients than in controls.^20^, ^21^, ^22^, ^25^, ^31^ We therefore investigated anti‐α‐Gal IgG and IgG subclass levels in sensitized and non‐sensitized FE. Anti‐α‐Gal IgG, IgG1, IgG2 and IgG3 levels were significantly higher in sensitized individuals, whereas α‐Gal IgG4 levels were very low in both groups (Figure 2). Since no significant difference in daily activities and exposure to tick bites could be seen between sensitized and non‐sensitized employees, a difference in IgG levels likely points towards a difference in immune reactions to tick bites in these groups.

### 3.3 AGS patients have higher levels of anti‐α‐gal IgG_1–3_ antibodies than sensitized FE

**FIGURE 3:**
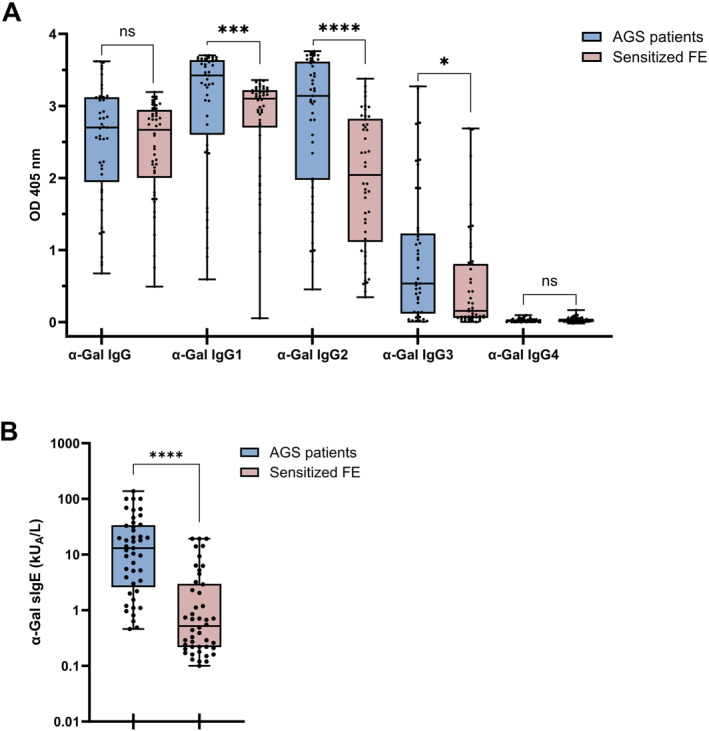
AGS patients have higher levels of IgG1 and IgG2 to α‐Gal‐HSA compared to sensitized FE. (A) Box‐plot showing levels of IgG and IgG subclasses directed towards α‐Gal in AGS patients (*n* = 45) and in sensitized FE (*n* = 46), multiple Mann‐Whitney test showing significance of comparison (ns: not significant, **p < 0.01, ****p < 0.0001). (B) α‐Gal‐specific IgE levels (kUᴀ/L) in AGS patients (*n* = 45) and sensitized FE (*n* = 46) as determined in ImmunoCAP. Mann‐Whitney test comparison (****p < 0.0001). Whiskers extend to minimum and maximum values, median displayed as a line within each box. FE, forestry employees.

Having found highly significant differences in levels of IgG and IgG subclasses between sensitized and non‐sensitized individuals, we also analyzed the anti‐α‐Gal IgG_1–4_ patterns among AGS patients (Table S1) and compared them to the IgG profiles of sensitized FE. AGS patients had significantly higher anti‐α‐Gal IgG1 and IgG2 levels than the sensitized FE (Figure 3A). Moreover, sIgE levels were significantly higher in AGS patients (Figure 3B). sIgE levels were moderately correlated with IgG1 levels in sensitized FE (*r* = 0.3505; *p* = 0.0170) and to IgG2 levels in AGS patients (*r* = 0.4168; *p* = 0.0044) (Figure S1).

### 3.4 AGS patients have a different IgG antibody profile than fish‐allergic patients

**FIGURE 4:**
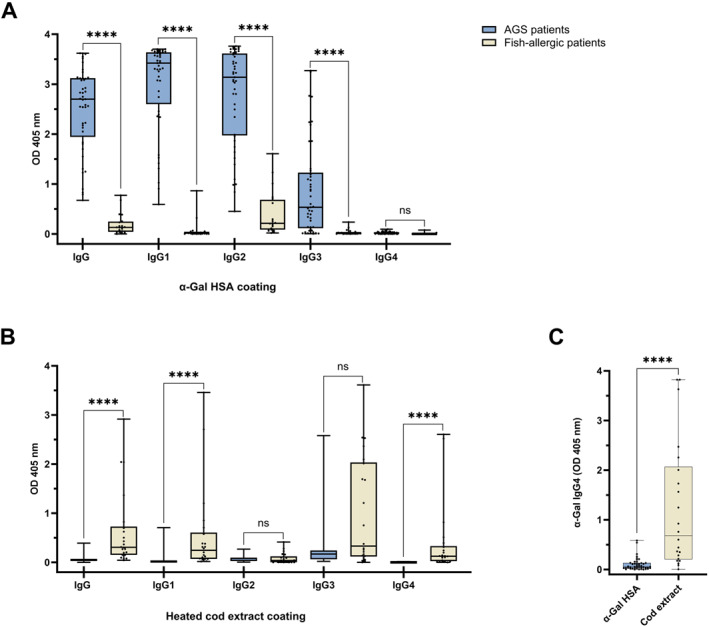
AGS patients have a significantly different allergen‐specific IgG profile than fish‐allergic patients. Box plot comparing IgG and IgG subclass levels directed against—(A) α‐Gal in AGS patients (*n* = 45) and fish‐allergic patients (*n* = 20), (B) heated cod extract in AGS patients (*n* = 25) and fish‐allergic patients (*n* = 22). Multiple Mann–Whitney comparison of antibody levels between groups (****p < 0.0001, ns: not significant). (C) Due to the low abundance of IgG4, a more sensitive assay was used to specifically compare IgG4 levels among patients using lower serum dilution, overnight incubation and an amplifying detection system. Mann–Whitney test showing significance of difference (****p < 0.0001). Whiskers extend to minimum and maximum values, median displayed as a line within each box.

In order to compare the IgG profiles of the first two cohorts, the high‐risk population of FE and the AGS patients, to a third cohort of patients with an unrelated food allergy, we analyzed the sera of a group of fish‐allergic patients (Table S2). In the first step, we compared AGS patients with fish‐allergic patients. IgG and IgG subclass profiles against α‐Gal and a heated cod extract, containing native parvalbumin as the main allergen component,^32^ were compared between both patient groups (Figure 4). In the first experiment using α‐Gal HSA coating, modest levels of IgG and IgG2 to α‐Gal were detected in fish‐allergic patients, whereas AGS patients had significantly higher levels of antibodies of all subclasses, except IgG4 (Figure 4A).

In a second experiment, with cod extract coating, we determined IgG and IgG subclass levels against cod in both patient groups (Figure 4B). As expected, fish‐allergic patients showed higher IgG responses to cod than AGS patients; however, highly significant differences were observed only in cases of IgG, IgG1 and IgG4. Lastly, using a high sensitivity assay for the detection of IgG4, we confirmed a very low IgG4 response to the carbohydrate α‐Gal in AGS patients compared to high IgG4 levels to protein allergens in fish‐allergic patients (Figure 4C). Although the age difference in both cohorts could play a role in antibody levels, our results are in line with other studies comparing IgG responses to α‐Gal to IgG directed to the major apple allergen Mal d 1 or major salmon allergen Sal s 1.^20^, ^22^ Furthermore, IgG, IgG1 and IgG2 levels to α‐Gal were much higher in AGS patients than their respective antibodies to cod in fish‐allergic patients (Figure S2).

### 3.5 Forest service employees develop a strong IgG response to α‐gal compared to control subjects

**FIGURE 5:**
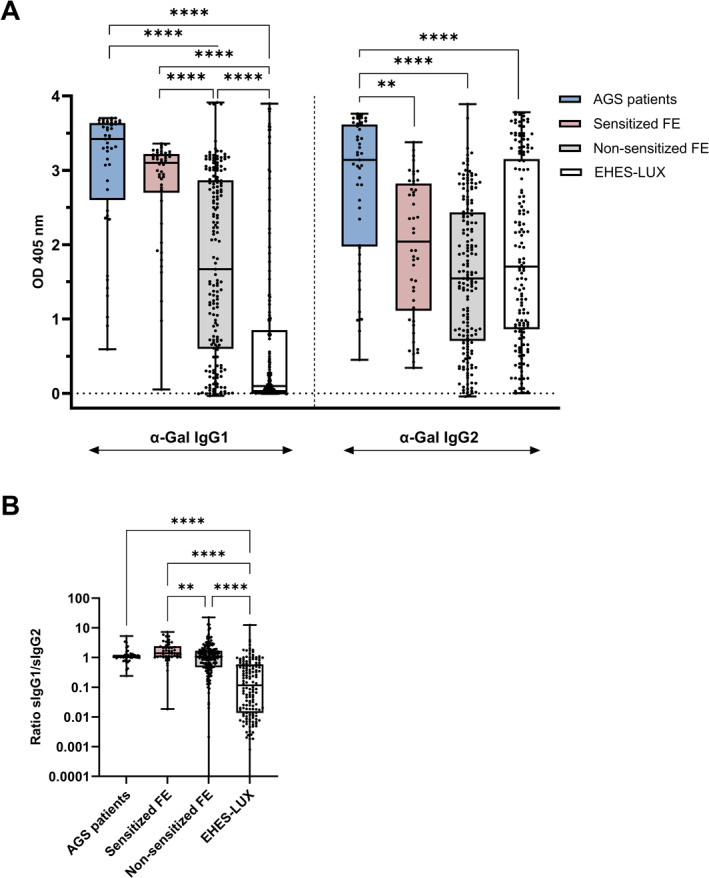
IgG1 and IgG2 levels to α‐Gal vary significantly across groups. (A) Box plot showing α‐Gal IgG1 and IgG2 levels in AGS patients (0.49 – >100 kUᴀ/L, *n* = 45), sensitized FE (sIgE ≥ 0.1 kUᴀ/L, *n* = 46), non‐sensitized FE (sIgE < 0.1 kUᴀ/L, *n* = 173), and EHES‐LUX controls (<0.1 – 0.68 kUᴀ/L; *n* = 150). Kruskal‐Wallis with multiple comparison test (**p < 0.01, ****p < 0.0001). (B) The ratio of sIgG1/sIgG2 is low in controls with low tick exposure. The ratio of OD_405nm_ values between sIgG1 and sIgG2 was plotted for AGS patients (*n* = 45), sensitized FE (*n* = 46), non‐sensitized FE (*n* = 173), and EHES‐LUX controls (*n* = 150). Kruskal‐Wallis with multiple comparison test (**p < 0.01, ****p < 0.0001). Whiskers extend to minimum and maximum values, median displayed as a line within each box. FE, forestry employees.

Finally, we compared IgG and IgG subclasses among the following groups: AGS patients, sensitized FE, non‐sensitized FE, and controls from EHES‐LUX, a population‐based cohort (Figure 5A; Figure S3). In this control population, the α‐Gal sensitization rate was 1.3% (2/150; α‐Gal sIgE 0.21 and 0.68 kU/L). AGS patients and sensitized FE had the highest α‐Gal sIgG and sIgG1 levels, followed by non‐sensitized FE, whereas control subjects showed quite low levels of IgG and IgG1 to α‐Gal (Figure 5A, Figure S3). AGS patients also had the highest IgG2 levels compared with FE and controls. The ratio of IgG1 to IgG2 antibody levels illustrates the relatively high abundance of IgG1 antibodies compared with IgG2 antibodies in AGS patients and FE in contrast to a low ratio in controls (Figure 5B). Whereas the α‐Gal IgG2 levels are not significantly different between non‐sensitized FE and controls, IgG1 levels are strikingly higher in non‐sensitized FE, suggesting a continuous stimulation of IgG1 immune responses to α‐Gal by repeated tick bites. In line with this observation, FEs reporting tick bites during the year of sample collection had significantly higher levels of IgG1 and IgG2 antibodies to α‐Gal compared with employees not recalling a history of tick bites (Figure S4). Levels of α‐Gal sIgG3 were lower than IgG1 and IgG2 levels; they were most abundant in AGS patients and sensitized FE (Figure S3). Although levels of IgG4 were statistically lower in controls than in tick bite exposed subjects, the main finding is the confirmation of characteristically low α‐Gal IgG4 levels in all groups.

## 4 DISCUSSION

The α‐Gal syndrome is considered an allergic disease that is mediated by tick bites. Ticks have been repeatedly shown to contain the α‐Gal epitope,^10^, ^33^, ^34^ although the source of the carbohydrate, either endogenously produced or originating from residual host blood, is still a matter of debate. sIgE directed at α‐Gal are associated with tick bites^11^ and higher sensitization rates are reported in tick‐endemic regions and in rural populations as well as in individuals with an occupational outdoor activity such as FE.^13^, ^14^, ^15^, ^16^, ^17^, ^30^, ^35^

In the present study, we retrospectively measured sIgE levels in a high‐risk cohort consisting of 219 forest service employees. Using a cutoff of >0.1 kU_A_/L, we found a sensitization rate of 21%. sIgE levels were low, which is in line with previous findings in high‐risk populations. The sensitization rate in FE in Luxembourg is lower than in southwest Germany and Kentucky, 35% and 39% respectively,^13^, ^14^ but higher than in FE in Spain (15%).^16^ The prevalence of anti‐α‐Gal sIgE in the European general population varies from 1.2% to 8.1%, based on a cutoff of 0.1 kU_A_/L and depending on regional differences and recreational activities.^15^, ^16^, ^36^, ^37^ In our control population, we found a prevalence of 1.3%, which lies in the lower range. Our results confirm previous findings of higher prevalence rates in populations with dominant perannual characteristics of outdoor activity. Sensitized individuals were found to have higher total IgE levels, which were correlated with α‐Gal IgE. This finding confirms data from previous studies where high total IgE levels were associated with α‐Gal sensitization.^13^, ^18^ High total IgE was also proposed to be a direct consequence of tick bites,^38^ but in our cross‐sectional study, we could not find a significant association of total IgE with the number of tick bites.

The main objective of our study was to analyze α‐Gal IgG and IgG subclass profiles in a high‐risk cohort and to compare them with the IgG profiles of AGS patients, patients with a non α‐Gal‐related food allergy and a cohort of control subjects age‐matched to the high‐risk cohort. Although previous studies have analyzed α‐Gal IgG and IgG subclass profiles in AGS patients and healthy controls,^20^, ^21^, ^22^, ^25^ our study is the first to investigate IgG responses to α‐Gal in a high‐risk population of FE. Sensitized as well as non‐sensitized FE had significantly higher levels of anti‐α‐Gal IgG1 and IgG3 antibodies than controls. Furthermore, employees with sIgE to α‐Gal were found to have significantly higher levels of α‐Gal IgG, IgG1, IgG2 and IgG3 than non‐sensitized participants. IgG2 levels were high in FE and controls and showed no significant difference between groups. The very low levels of α‐Gal IgG1 found in controls compared to non‐sensitized, but tick exposed FE, argue in favor of a stimulation of an IgG1 response on the background of an existing IgG2 response. These findings are in line with previous studies showing high IgG1 responses to α‐Gal in AGS patients compared with controls,^20^, ^21^, ^22^, ^25^ but our study is the first to confirm high IgG1 levels to α‐Gal in non‐sensitized individuals with recurrent tick bites. Tick saliva contains immunomodulatory molecules which are known to shift the immune response to a Th2 type.^39^ Consequently, high levels of inflammatory IgG1 antibodies are expected upon repeated tick bites and a Th2 environment would promote a direct switch from IgG1 to IgE antibodies.

Notably, fish‐allergic patients displayed an antibody profile that was similar to the profile of the controls: IgG2 antibodies against α‐Gal, but no relevant IgG1 or IgG3 responses. Dominant IgG2 responses to carbohydrates in general^40^, ^41^ and specifically to α‐Gal have been found earlier^24^, ^42^ and have been confirmed in blood donors and non‐allergic controls.^20^, ^21^, ^22^, ^23^, ^25^ Anti‐α‐Gal IgG are hypothesized to originate from stimulation by gut microbiota,^5^ although the presence of α‐Gal on gut bacteria has recently been questioned.^43^

In a recent study by Joral et al, IgG against α‐Gal was quantified in AGS patients, in individuals bitten by ticks and in healthy controls. IgG was found to be elevated in AGS patients compared with subjects bitten by ticks, and the quantification of IgG against α‐Gal was proposed as a prognostic marker for developing α‐Gal allergy.^31^ Although in our study IgG to α‐Gal was highest in AGS patients and sensitized FE, we also found significantly increased levels of IgG in non‐sensitized FE compared with controls. Many employees were employed for more than 10 years without developing a sensitization to α‐Gal, despite having high IgG levels.

Another important finding of our study is that fish‐allergic patients showed a strong IgG4 response to fish extract, whereas α‐Gal specific IgG4 levels in sensitized FE and AGS patients were barely above the detection level. This finding is in accordance with previous studies comparing IgG4 responses to α‐Gal in AGS patients with IgG4 directed at Mal d 1 in patients with birch pollen‐related apple allergy^20^ and at salmon parvalbumin in fish‐allergic patients.^22^ Thus, allergic as well as non‐allergic responses to α‐Gal are characterized by the absence of specific IgG4 antibodies. The finding that no IgG4 antibodies were detectable in a tolerant but exposed population is novel, and warrants further investigation on immune responses directed at α‐Gal and carbohydrates in general. Our observation has an important implication in the assessment of immunotherapy success or natural resolution of the α‐Gal syndrome as the presence of IgG4 is often a marker of tolerance induction in protein‐mediated food allergy. In contrast, in exposed beekeepers, venom‐specific IgG4 levels are positively related to the number of years working in the apiary and the number of received bee stings.^44^, ^45^ Another important observation relates to the fact that in AGS patients and in FE, IgG antibody levels directed at α‐Gal are significantly higher than IgG directed at fish extract in fish‐allergic patients (except for IgG4). In patients with sIgE to CCD, IgG responses were also dominated by IgG1, although levels were lower than IgG1 against α‐Gal in AGS patients.^22^ Whether these high antibody levels are a general characteristic for α‐Gal related IgG responses remains to be confirmed.

A major strength of our study is the analysis of anti‐α‐Gal IgG and IgG subclass profiles in a large cohort of α‐Gal sensitized and non‐sensitized participants that have a high occupational risk of recurrent tick bites. The comparison of these profiles with control subjects and food‐allergic patients, a group allergic to α‐Gal and a group allergic to fish, allowed us to discover distinct IgG profiles in individuals bitten repeatedly by ticks. A weakness of our study is certainly the lack of clinical data of the high‐risk population as well as data on mammalian meat consumption. A German study on FE detected a total of five individuals with AGS among the study group, representing 8.6% of the group with sIgE ≥0.35 kU_A_/L.^13^ When extrapolating to our cohort, about 2–3 individuals may potentially be affected by AGS. However, using the cutoff of 0.54 kU_A_/L established by ROC curve analysis in a large German cohort,^46^ 21 individuals would be labeled as potentially allergic to α‐Gal. Although the lack of clinical data is certainly a drawback of our retrospective study, we are confident that the presence of 2%–9% AGS patients among the 219 FEs, depending on the cutoff used for extrapolation, would not impact the major conclusions of our study.

In conclusion, our study shows elevated levels of IgG directed against α‐Gal in a high‐risk cohort of FE with recurrent tick bites. Whereas IgE‐sensitized employees present an IgG response dominated by IgG1, non‐sensitized individuals have a more equilibrated IgG1 and IgG2 response, which is in contrast to the very low IgG1 levels in controls. Our findings in non‐sensitized individuals exposed to multiple tick bites thus imply that α‐Gal IgG1 responses are induced by tick bites. A hallmark of sensitization to α‐Gal is the lack of a marked IgG4 response to α‐Gal, distinguishing α‐Gal allergy from protein‐related food allergies.

## AUTHOR CONTRIBUTIONS

**Neera Chakrapani**: Conceptualization; data curation; formal analysis; methodology; visualization; writing—original draft; writing—review & editing. **Kyra Swiontek**: Formal analysis; investigation; methodology; validation; visualization; writing—original draft; writing—review & editing. **Judith M. Hübschen**: Investigation; methodology; resources; supervision; writing—original draft; writing—review & editing. **Jörg Fischer**: Conceptualization; formal analysis; funding acquisition; investigation; resources; validation; writing—original draft; writing—review & editing. **Maria Ruiz‐Castell**: Data curation; resources; supervision; writing—review & editing. **Francoise Codreanu‐Morel**: Investigation; resources; validation; writing—review & editing. **Farah Hannachi**: Investigation; resources; validation; writing—review & editing. **Martine Morisset**: Investigation; resources; validation; writing—review & editing. **Markus Ollert**: Funding acquisition; supervision; validation; writing—review & editing. **Annette Kuehn**: Investigation; methodology; resources; supervision; validation; writing—review & editing. **Claude P. Muller**: Funding acquisition; resources; supervision; writing—review & editing. **Christiane Hilger**: Conceptualization; funding acquisition; methodology; resources; supervision; validation; writing—original draft; writing—review & editing.

## CONFLICT OF INTEREST STATEMENT

All authors declare no conflicts of interest.

## Supporting information

Supporting Information S1

## Data Availability

Additional data supporting the findings of this study are available on reasonable request from the corresponding author. The data are not publicly available due to privacy or ethical restrictions.
