# Supplementary material for: Recurrent tick bites induce high IgG1 antibody responses to α‐Gal in sensitized and non‐sensitized forestry employees in Luxembourg
Source: Clin Transl Allergy. 2024 Oct 13;14(10):e12396. doi: 10.1002/clt2.12396 (PMC11471575; doi:10.1002/clt2.12396)
Supplement: Supplementary file 1 — Supporting Information S1 [file CLT2-14-e12396-s001.pdf]

Recurrent tick bites induce high IgG1 antibody responses to α-Gal in sensitized and non-sensitized forestry employees in Luxembourg

Short title: Anti-α-Gal IgG$_{1\text{-}4}$ responses in forestry employees

Neera Chakrapani[1, 2], PhD, Kyra Swiontek[1], MSc, Judith M. Hübschen[1], PhD, Jörg Fischer[3 **], MD, Maria Ruiz-Castell[4], PhD, Francoise Codreanu-Morel[5], MD, Farah Hannachi[5], MD, Martine Morisset[5 **], MD, PhD, Markus Ollert[1,6], MD, DMSci, Annette Kuehn[1], PhD, Claude P. Muller[1], MD, MS, Christiane Hilger[1 *], PhD

[1] Department of Infection and Immunity, Luxembourg Institute of Health, Esch-sur-Alzette, Luxembourg;

[2] Faculty of Science, Technology and Medicine, University of Luxembourg, Esch-sur-Alzette, Luxembourg;

[3] Department of Dermatology, Faculty of Medicine, Eberhard Karls University of Tübingen, Tübingen, Germany;

[4] Department of Precision Health, Luxembourg Institute of Health, Strassen, Luxembourg;

[5] The Immunology–Allergology Unit, Centre Hospitalier Luxembourg, Luxembourg

[6] Department of Dermatology and Allergy Center, Odense Research Center for Anaphylaxis, University of Southern Denmark, Odense, Denmark

*Corresponding author: Dr. Christiane Hilger, PhD

Department of Infection and Immunity, Luxembourg Institute of Health (LIH)

29, rue Henri Koch, Esch-sur-Alzette, L-4354, Luxembourg

Telephone: +352 26 970 258

E-mail: christiane.hilger@lih.lu

Present addresses:

N Chakrapani, ALK, Global Research, Hoersholm, Denmark.J Fischer, Department of Dermatology and Allergology, University Hospital, Augsburg, Germany.

M Morisset, Allergy Unit, Angers University Hospital, Angers, France.

SUPPLEMENTARY TABLE AND FIGURES

Table S1: Clinical characteristics of AGS patients

Count	Sex	Age (yr)	Trigger of anaphylactic reaction	Symptoms related to α-Gal	Positive skin tests (prick/ic)	α-Gal sIgE (kU_A/L)	tIgE (kU_A/L)	Tick bites
1	F	52	spare ribs (pork), lamb	U, D	P, B, PK, BK, G	14	443	n.d.
2	F	51	beef	U	P, B, PK, BK, G	5.1	125	yes (#)
3	M	37	beef kidney	U	P, B, PK, BK, G	0.81	122	no (#)
4	F	81	pork kidney	U, D, F	P, B, PK, BK, G	0.49	60.9	no (#)
5	F	53	milk, cheese	U, D, Ap	P, B, PK, BK, G	31.5	821	yes (#)
6	M	79	pork kidney	U, CS	P, B, PK, BK, G	37.4	678	n.d.
7	F	60	pork kidney	U, D	P, B, PK, BK, G	20.2	96.5	n.d.
8	F	64	pork, beef	U	P, B, PK, BK, G	3	115	n.d.
9	M	18	sausages	U	P, B, PK, BK, G	34.4	305	yes (#)
10	M	63	pork kidney	U	P, B, PK, BK, G	7.1	228	n.d.
11	M	59	pork kidney	U, CS, F	P, B, PK, BK, G	20.1	86.2	no (#)
12	M	56	pork kidney, pork, beef, deer, gelatin	U, D, CS, F	P, B, PK, BK, G	50.8	3142	no (#)
13	F	73	pork kidney, pork, beef	U, D	PK, G	2.2	8.7	no (#)
14	F	24	pork, beef	U	P, B	9.4	36.5	no (#)
15	M	70	pork, beef	U, F	PK, BK, B	46	800	no (#)
16	M	21	beef	U, Ap	PK , B	3.8	n.d.	yes
17	M	43	pork, beef, mutton	U, C, W, H	n.d.	138	562	n.d.
18	M	13	pork meat	U, Ap, V, HP	P, PK	69	765	yes (#)
19	M	19	sport	U, N, Ap	B, PK	5.52	80	yes (#)
20	F	29	ground meat, ham, sausage	U, palpebral AE	B, PK	1.55	72	n.d.
21	F	31	cheeseburger, cheese	U, lingual AE	B, P, PK	2	18	yes
22	M	46	ground meat, sausage, cheese	U	B, PK	18	32	yes (#)
23	M	48	sausages	U, V, D, labial or lg AE	B, P, PK	12	126	n.d.
24	M	20	ice cream, cheese, gelatin	n.d.	PK	1.2	168	n.d.
25	M	20	bolognese or cheese	Ap, V, F	PK	13	143	yes
26	F	52	dairy products	U	PK, M	0.96	42	n.d.
27	F	48	n.d.	U, palpebral AE	B, PK	21	190	yes (#)
28	M	62	i.v. infusion beef gelatin	PA	PK	18	4993	n.d.

29	M	27	beef meat, red meats, ex. with alcohol	U, D, W	B, P, PK	66	373	yes
30	M	32	beef and pork meat	U, D, C, lg Dy	B, P, PK	27	401	n.d.
31	M	56	red meat with ACE inhibitor	AE	PK, B	>100	>1000	yes
32	M	35	fat meal	LD	PK	0.63	205	yes
33	M	45	lamb, ham	U, P, mild Bs	PK, P, B	63	639	yes (#)
34	M	55	beef, ex. with alcool	N, U, S	PK, P, B, L, M	>100	19040	yes (#)
35	F	69	pork kidney, innards, ex. with alcool	Bs, U	PK, L,B, M	33	201	yes (#)
36	F	67	beef and pork meat	U	PK, BK, G	1.1	242	no (#)
37	M	54	pork kidney, innards	N, Ap	n.a.	1.1	267	yes (#)
38	M	63	pork kidney, innards	U, S	n.a.	0.46	115	yes (#)
39	F	80	beef and pork meat	U, S	P, B, PK, BK, G	3.4	129	no (#)
40	M	56	beef and pork meat	U	n.a.	19.7	365	no (#)
41	M	75	pork kidney	U	PK, BK	100	820	yes (#)
42	M	80	pork kidney	U	n.a.	5.2	102	yes (#)
43	M	75	innards	n.a.	n.a.	9.6	359	yes (#)
44	M	63	pork kidney, innards	U	n.a.	17.1	101	yes (#)
45	F	65	pork kidney	U	PK, BK, G	10.4	30.8	yes (#)
Median		54				12	190	
Range		13-81				0.46 - >100	8.7 - 19040	

n.a.: not applicable
n.d.: no data available
# : within 12 months prior sampling
ex.: exacerbation

Symptoms - AE: angioedema, Ap: abdominal pain, Bs: bronchospasm, C: cough, CS: circulatory shock, D: dyspnea, Dy: dysphagia, F: faintness, H: hypotension, HP: heart palpitations, lg: laryngeal, N: nausea, P: pruritus, PA: perioperative anaphylaxis, S: syncope, U: urticaria, V: vomiting, W: wheezing.

Skin prick tests - B: beef meat, BK: beef kidney, G: gelatin, L: lamb, M: milk, P: pork meat, PK: pork kidney.

Table S2: Basic clinical and demographic data of cohorts

	**AGS**	**Fish-allergic**	**Sensitized FE**	**Non-sensitized FE**	**EHES-LUX controls**
No. (N)	45	22	46	173	150
Sex (male%)	29/45 (64%)	18/22 (82%)	45/46 (98%)	166/173 (96%)	62/150 (41%)
Age (yrs)	54 (13-80)	12 (7-35)	43 (19-63)	45 (19-63)	45 (26-64)
sIgE (kUA/L)	12 (0.46- >100)	10.40 (6.5- >100)	0.52 (0.1-19.3)	< 0.1	< 0.1 (<0.1-0.68)
Total IgE (kU/L)	190 (8.7-19040)	828 (48-2297)	271.5 (26.5-2844.4)	78 (0-2115.1)	n.d.

sIgE values are against α-Gal in AGS patients, sensitized and non-sensitized FE and against cod protein in fish-allergic. N, number; yrs, years; FE, forestry employees; n.d., not determined; age/sIgE/total IgE, values are given as median with range

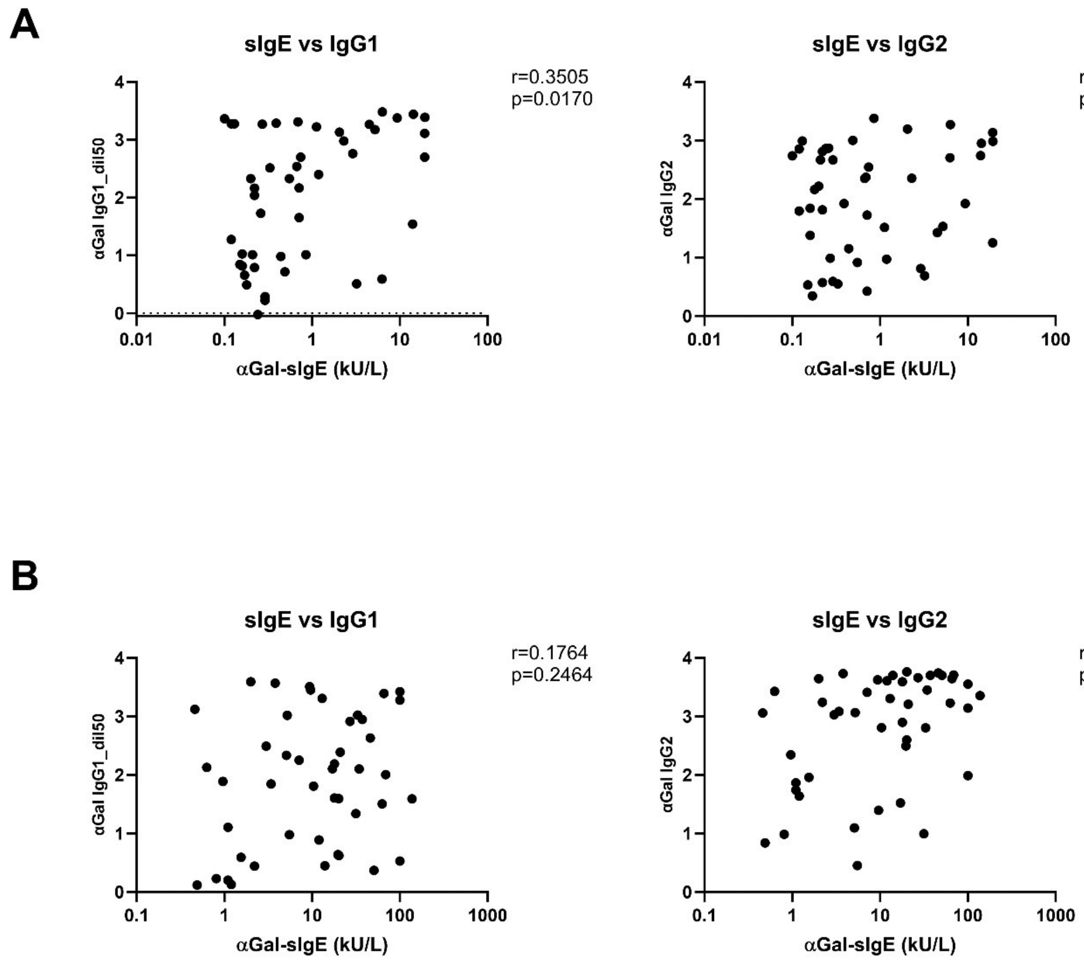

Fig. S1: Correlations of IgG and IgE antibody responses to α-Gal in participants sensitized to α-Gal. A. Correlation of IgG1, respectively IgG2, and sIgE in sensitized FE. B. Correlation of IgG1, respectively IgG2, and sIgE in AGS patients. Spearman r analysis.

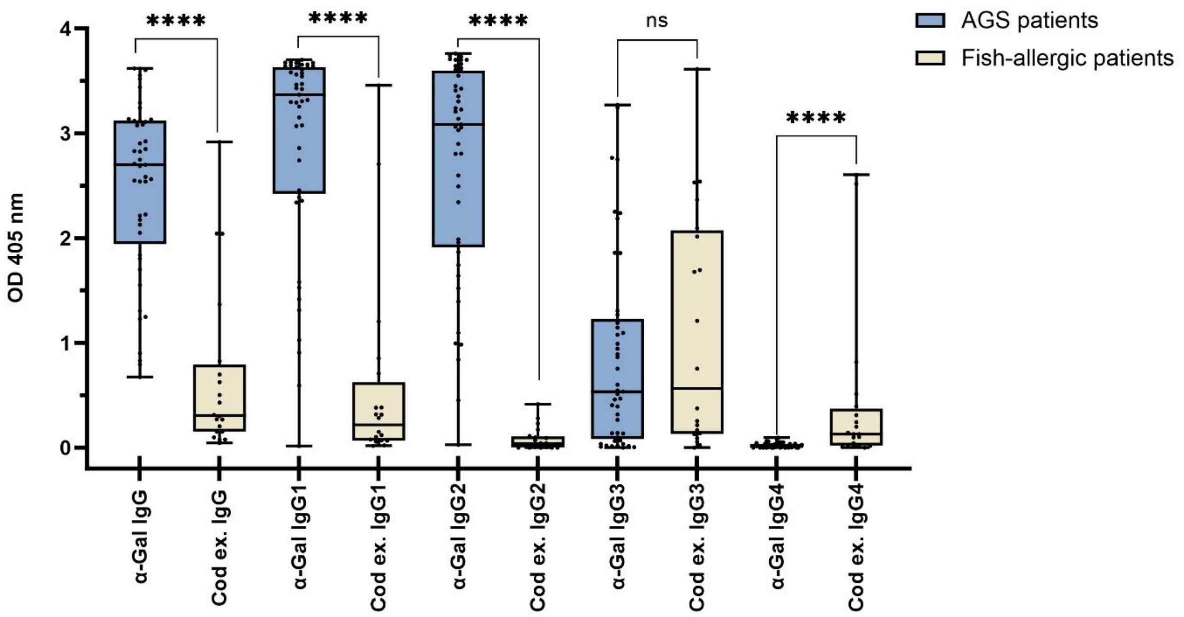

Fig S2. AGS patients have significantly higher IgG levels to α-Gal compared to fish-allergic patients to cod extract. Box plot showing IgG/subclass levels in AGS patients to α-Gal HSA (n=45) and IgG/subclass levels to cod extract in fish-allergic patients (n=22). Multiple Mann-Whitney comparisons show significant differences in IgG/subclass levels between groups to the respective allergen/extract (**** p<0.0001, ns: not significant).

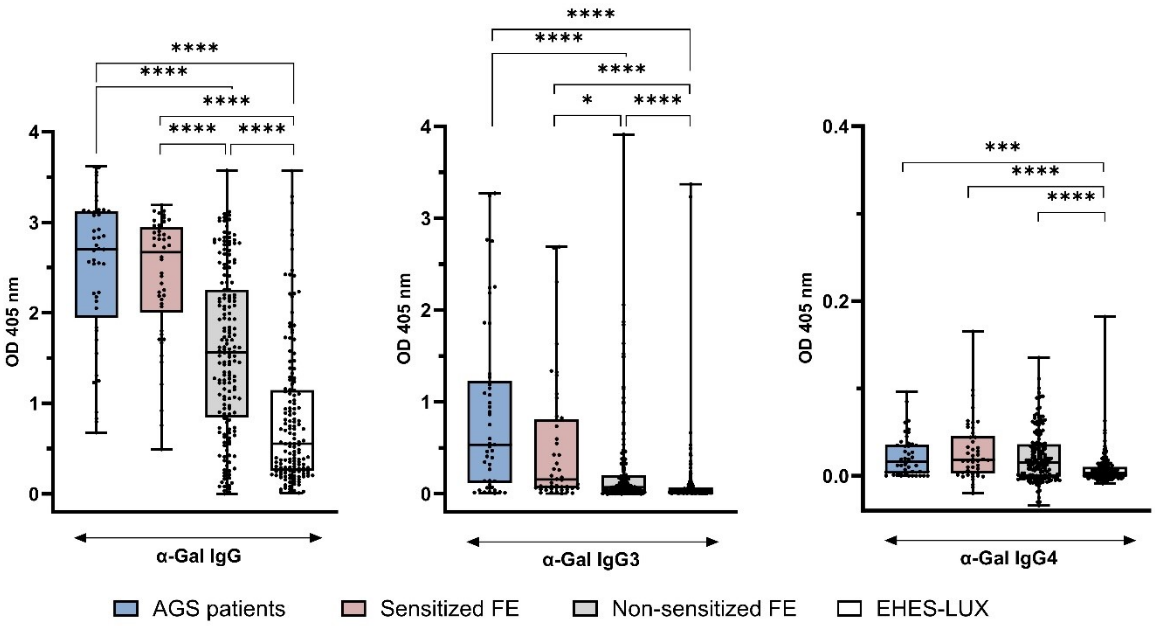

Fig S3. IgG, IgG3 and IgG4 levels to α-Gal are significantly different across groups. Box plot showing α-Gal IgG3 and IgG4 levels in AGS (0.49 – >100 kUᴀ/L, n=45), sensitized FE (sIgE ≥0.1 kUᴀ/L, n=46), non-sensitized FE (sIgE <0.1 kUᴀ/L, n=173), and EHES controls (<0.1– 0.68 kUᴀ/L; n=150). Kruskal-Wallis with multiple comparison test (* p<0.05, *** p<0.001, **** p<0.0001). Whiskers extend to minimum and maximum values, median displayed as line within each box.

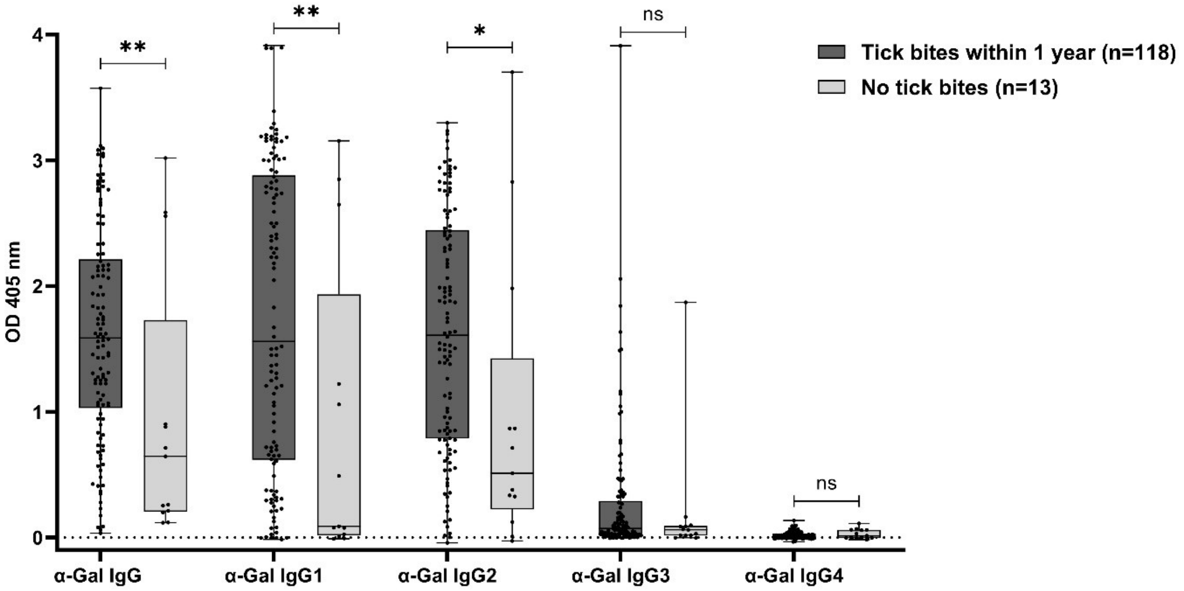

Fig S4. α-Gal IgG/subclass levels in non-sensitized FE recalling no tick bites ever (n=13) versus those bitten within one year before sample collection (n=118). Multiple Mann-Whitney comparisons show significant differences in α-Gal IgG, IgG1, and IgG2 levels (** $p<0.01$, * $p<0.05$, ns: not significant).
